# Accuracy of Procalcitonin Levels for Diagnosis of Culture-Positive Sepsis in Critically Ill Trauma Patients: A Retrospective Analysis

**DOI:** 10.7759/cureus.12988

**Published:** 2021-01-29

**Authors:** Aisha Bakhtiar, Syed Jawad Haider Kazmi, Muhammad Sohaib Asghar, Muhammad Nadeem Khurshaidi, Salman Mazhar, Noman A Khan, Nisar Ahmed, Farah Yasmin, Rabail Yaseen, Maira Hassan

**Affiliations:** 1 Pediatric Surgery, Liaquat National Hospital, Karachi, PAK; 2 Emergency Medicine, Liaquat National Hospital, Karachi, PAK; 3 Internal Medicine, Dow University of Health Sciences, Karachi, PAK; 4 General Surgery, Liaquat National Hospital, Karachi, PAK; 5 Cardiothoracic Surgery, Aga Khan University Hospital, Karachi, PAK; 6 Internal Medicine, Liaquat National Hospital, Karachi, PAK

**Keywords:** trauma, general trauma surgery, elderly trauma, trauma pediatric, sepsis treatment, severe sepsis, organ failure from sepsis, blood culture, procalcitonin, diagnostic test accuracy

## Abstract

Background

Abdominal trauma and intra-abdominal sepsis are associated with significant morbidity and mortality. Microcirculation in the gut is disrupted in hemorrhagic and septic shock leading to tissue hypoxia, and the damaged gut acts as a reservoir rich in inflammatory mediators and provides a continual source of inflammation to the systemic circulation leading to sepsis. Sepsis is defined as the presence (probable or documented) of infection together with a systemic inflammatory response to infection. Blood culture is commonly considered to be the preferred approach for diagnosing sepsis, although it is time-consuming, that is, reports are normally available only after 12-48 hours. Procalcitonin levels (PCT) have recently emerged as a promising biomarker in the diagnosis of sepsis. The aim of the present study is to determine the diagnostic accuracy of PCT levels in predicting sepsis in critically ill trauma patients.

Methodology

This was designed as a validation study conducted in the Indoor Department of General Surgery, Liaquat National Hospital, Karachi. The sample size was calculated by taking the estimated frequency of sepsis in suspected patients at 62.13%, expected sensitivity of PCT at 70.83%, and specificity at 84.21% and the desired precision level of 12% for sensitivity; the calculated sample size was 96. The non-probability consecutive sampling method was used to recruit participants who were diagnosed with sepsis on clinical assessment. Blood culture samples were sent for the enrolled patients and a final diagnosis was made on the blood culture report. PCT levels were measured in these suspected patients on the same day of sending blood culture. Diagnostic accuracy of PCT size was measured using the receiver operating characteristic (ROC) curve. ROC curve was formulated for PCT levels against culture-proven sepsis to determine the ideal cut-off value of PCT levels. Two different cut-offs were determined to obtain the highest sensitivity and highest specificity accordingly.

Results

A total of 97 individuals met the inclusion criteria with a mean age of 34.89 ± 10.52 years. Mean PCT levels were 0.96 ± 0.59, with a gender predilection towards females (p < 0.001). No age difference was documented among gender (p = 0.655). The mean duration of intensive care unit stay was 11.73 ± 3.56 days. Culture-proven sepsis was identified in 67.0% of the study participants with a higher PCT level (p < 0.001). Among the 52.6% males included in the study, half were reported to have culture-positive sepsis, but among the 47.4% females culture was positive in 87% (p < 0.001). ROC revealed PCT was predictive for culture-positive sepsis at a cut-off value 0.47 ng/mL (p < 0.001), with a sensitivity of 92.3%, specificity of 68.7%, positive predictive value (PPV) of 85.7%, and negative predictive value (NPV) of 81.5%. By increasing the cut-off value to 0.90 ng/mL at area under the curve of 0.816, the specificity increased to 81.3% and sensitivity became 66.2%, with a PPV of 87.8% and NPV of 54.2%.

Conclusion

Our study determined two cut-values for PCT to predict sepsis, one with the highest sensitivity and the other with better specificity. Other than that, higher PCT levels were significant in female trauma patients. We conclude that PCT is a reliable marker for culture-proven diagnosis of sepsis and may aid physicians/surgeons to promptly manage patients accordingly.

## Introduction

Abdominal trauma and intra-abdominal sepsis are associated with significant morbidity and mortality [1,2]. Microcirculation in the gut is disrupted in hemorrhagic and septic shock leading to tissue hypoxia, disruption of barrier integrity, increased mucosal permeability, release of pro-inflammatory mediators, and ascites. The damaged gut acts as a reservoir rich in inflammatory mediators and provides a continual source of inflammation to the systemic circulation and leads to sepsis. Sepsis is likely the initial motor of multiple organ dysfunction (MOD) [3,4]. A study by Chung et al. reported that sepsis occurs in 11.85% of trauma patients [5].

Sepsis is defined as the presence (probable or documented) of infection together with a systemic inflammatory response to infection. It is a life-threatening disease that causes millions of deaths globally each year [6]. Blood culture is commonly considered to be the preferred approach for diagnosing sepsis, although it is time-consuming, that is, reports are normally available only after 12-48 hours. In addition, skin contamination may mislead physicians in some cases [7]. As a result, because the ambiguous omission of sepsis from the differential diagnosis list in acute stages is undesirable, the use of empiric antibiotics is typically unavoidable and has untoward consequences [8].

Procalcitonin (PCT) levels have recently emerged as a promising biomarker in the diagnosis of sepsis. PCT levels rapidly rise (6-12 hours) after infection occurs. A recent study by Mustafić et al. reported that PCT is a valuable biomarker for the early detection of sepsis in suspected patients. They reported that PCT at a cut-off value 0.57 ng/mL is 97.56% sensitive and 95.83% specific for the diagnosis of sepsis [9]. Another study by Ahmed et al. reported that PCT level at 0.5 ng/mL is 93.75% sensitive and 43.59% specific for predicting sepsis in critically ill patients. They diagnosed sepsis in 62.13% of suspected patients [10].

The aim of the present study is to determine the diagnostic accuracy of PCT levels in predicting sepsis in critically ill trauma patients. PCT is an old test but there is still a debate on its diagnostic accuracy and in deciding the ideal cut-off value of PCT for predicting sepsis. Early identification of patients at risk of developing posttraumatic complications is crucial to allow the provision of early and appropriate therapy for sepsis.

## Materials and methods

This study was designed as a validation study conducted in the Indoor Department of General Surgery, Liaquat National Hospital, Karachi. The sample size was calculated by taking the estimated frequency of sepsis in suspected patients at 62.13%, expected sensitivity of PCT at 70.83%, and specificity at 84.21% and the desired precision level of 12% for sensitivity; the calculated sample size was 96. The non-probability consecutive sampling method was used to recruit the participants. The data included patients of trauma (both genders) admitted to the intensive care unit (ICU) in the last six months with a suspicion of sepsis (between ages of 12 and 70 years). The included patients with trauma had damage to vital organs necessitating admission to the ICU. The patients had clinical suspicion of sepsis such as temperature ≥38 °C or <36 °C, heart rate >90 beats/min, respiratory rate >20 breaths/min, and white blood cell count <4 × 10^9^/L (<4,000/mm³) or >12 × 10^9^/L (>12,000/mm^3^). Blood culture samples were sent for the enrolled patients and a final diagnosis was made on the blood culture report. PCT levels were measured in these suspected patients on the same day of sending blood culture. Patients above 70 years of age were excluded due to “immune senescence” in the elderly can give rise to different presentations of bacteremia.

Data regarding patients’ demographics such as age, gender, and duration of ICU stay were collected for each patient from medical records. Data were entered and analyzed using Statistical Package for Social Sciences version 25.0 (IBM Corp., Armonk, NY, USA). Mean and standard deviation were reported for quantitative variables such as age, duration of ICU stay, and PCT levels. Qualitative variables such as gender and sepsis on culture reporting were presented as frequency and percentage. Diagnostic accuracy of PCT size was measured using the receiver operating characteristic (ROC) curve. ROC curve was formulated for PCT levels against culture-proven sepsis to determine the ideal cut-off value of PCT levels. The ideal cut-off value of PCT was determined using area under the curve (AUC), and sensitivity, specificity, positive predictive value (PPV), and negative predictive value (NPV) were calculated against the cut-off value. Two different cut-offs were determined to obtain the highest sensitivity and highest specificity. Subsequently, a 2 × 2 contingency table was formulated to determine the sensitivity, specificity, PPV, and NPV of PCT levels taking culture reporting as the gold standard.

## Results

A total of 97 individuals met the inclusion criteria with a mean age of 34.89 ± 10.52 years. Mean PCT levels were 0.96 ± 0.59 ng/mL, with a gender predilection towards females (p < 0.001). No age difference was documented among gender (p = 0.655). The mean duration of ICU stay was 11.73 ± 3.56 days. Culture-proven sepsis was identified in 67.0% of the study participants with a higher PCT level (p < 0.001). Among the 52.6% males included in the study, half were reported to have culture-positive sepsis, but among the 47.4% females, culture was positive in 87% (p < 0.001), as shown in Table 1.

**Table 1:** Descriptive data of the study population (n = 97). PCT: procalcitonin; ICU: intensive care unit * values calculated by independent sample t-test. ^†^ values calculated by Chi-square test.

Variables	Frequency/Descriptives		P-Value
Age (years)	34.89 ± 10.52		-
PCT (ng/mL)	0.96 ± 0.59		-
Duration of ICU stay (days)	11.73 ± 3.56		-
Gender	Males: 51 (52.6%)	Females: 46 (47.4%)	-
	Mean age: 35.35 ± 10.26	Mean age: 34.39 ± 10.89	0.655*
	PCT: 0.47 ± 0.27	PCT: 1.51 ± 0.30	<0.001*
Sepsis on culture	Positive: 65 (67.0%)	Negative: 32 (33.0%)	-
	Males: 25 (38.5%)	Males: 26 (81.3%)	<0.001^†^
	Females: 40 (61.5%)	Females: 6 (18.7%)	
	PCT: 1.17 ± 0.53	PCT: 0.53 ± 0.47	<0.001*

**Figure 1:**
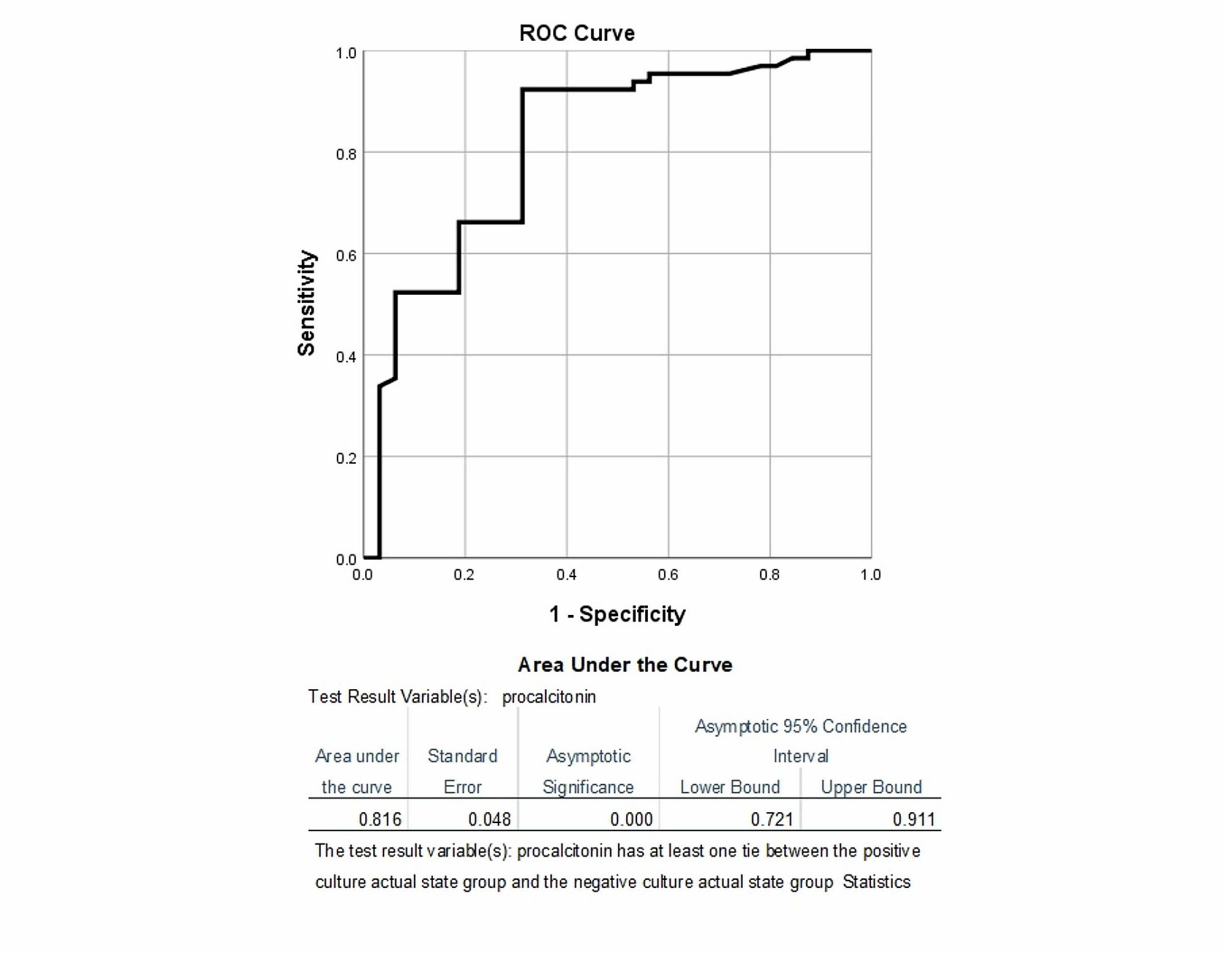
ROC curve for PCT predicting culture-positive sepsis. ROC: receiver operating characteristics; PCT: procalcitonin

ROC revealed PCT was predictive for culture-positive sepsis at a cut-off value 0.47 ng/mL (p < 0.001), with a sensitivity of 92.3%, specificity of 68.7%, PPV of 85.7%, and NPV of 81.5%. By increasing the cut-off value to 0.90 ng/mL at AUC of 0.816, the specificity increased to 81.3%, sensitivity became 66.2%, with a PPV of 87.8% and NPV of 54.2%, as shown in Figure 1.

## Discussion

Critical traumatic injuries frequently give rise to a quick large systemic inflammation and delayed immune responses, dysregulating immune system homeostasis and subjecting sufferers to sepsis and its manifestations with an eventual calamitous outcome [4]. In recent years, multiple studies have been regulated in detecting the role of inflammatory mediators in diagnosing sepsis in severely ill trauma patients. In miscellaneous studies, the mean age of significantly ill traumatic patients suffering from sepsis ranged from 40 to 71 years, making it prevalent in the middle-aged and elderly population [4,8,10,11]. Sepsis in sufferers of trauma displayed genetic affinity towards males compared to females [8,10,11].

In numerous studies, levels of PCT, C-reactive protein (CRP), neutrophil-lymphocyte ratio (NLR), and total leukocyte count (TLC) were significantly elevated in patients with positive bacterial growth cultures compared to negative cultures [4,8,11]. *Escherichia coli *and *Staphylococcus aureus* were prominently isolated organisms within cultures reported by various studies [4,8]. In a study conducted by Zheng et al., the accuracy of inflammatory markers in diagnosing sepsis was compared within patients suffering from hospital-acquired pneumonia (HAP), and among those with no infection, the levels of PCT, CRP, NLR, and TLC were raised prominently in patients with HAP along with frequent admissions in ICUs and comorbidities, while the increased frequency of malignancies was suffered by patients with no infections. The prevalence of surgery and 28-day survival rate was higher in patients with no infection while the mortality rate was significant in patients with HAP. Elevated PCT levels and other inflammatory mediators were detected in non-survivors, proving that inflammatory markers predict mortality in critically ill patients. Nonetheless, serum lactate levels and neutrophil count did not project striking differences in both groups [11]. In another study regulated between the middle-aged and elderly population, urinary tract infections and respiratory infections were prominent causes of sepsis. No significant difference in PCT levels was observed in both genders, within the adult and elderly population, while a prominent elevation in PCT levels of both adult and the elderly group was observed within non-survivors compared to survivors, supporting the outcome of the study mentioned above [8]. The specificity and sensitivity of PCT levels were slightly higher in the adult group compared to the elderly group [8].

Our study determined two cut-off values for PCT to predict sepsis, one with the highest sensitivity of 92%, and the other with a better specificity of 81.3%. Other than that, a higher PCT level was significant in female trauma patients as opposed to no significant difference among gender according to another study [8]. There were many limitations in our study, including a single-center design, limited number of patients, and lack of randomization in the study. Cost-benefit analysis and outcome analysis would have also shown the utility of PCT. Also, PCT is sensitive to bacterial sepsis and is not reliable in other sepsis (fungal). Furthermore, the non-specific nature of the increase in PCT in severe trauma has to be taken into account. As gold standard (blood culture) was the primary variable, the inclusion of outcome, cost-benefit, comparison of other markers (NLR, CRP, etc.) would have given more weight to the analysis, which was missing from our data.

## Conclusions

We conclude that PCT was proven to be a reliable marker for culture-proven diagnosis of sepsis and may aid physicians/surgeons to promptly manage patients accordingly. Previous studies have shown that prompt and effective sepsis treatment minimize MOD, decrease mortality, and improve clinical outcomes. Any examination or clinical information that facilitates early detection or safely prompts timely, effective sepsis treatment can save lives.
